# Mild Traumatic Brain Injury Evokes Pyramidal Neuron Axon Initial Segment Plasticity and Diffuse Presynaptic Inhibitory Terminal Loss

**DOI:** 10.3389/fncel.2017.00157

**Published:** 2017-06-06

**Authors:** Michal Vascak, Jianli Sun, Matthew Baer, Kimberle M. Jacobs, John T. Povlishock

**Affiliations:** Department of Anatomy and Neurobiology, Virginia Commonwealth University School of MedicineRichmond, VA, United States

**Keywords:** mild traumatic brain injury, axon initial segment, GABAergic synapse, plasticity, action potential initiation, layer 5 pyramidal neurons, transgenic mouse models, excitation-inhibition balance

## Abstract

The axon initial segment (AIS) is the site of action potential (AP) initiation, thus a crucial regulator of neuronal activity. In excitatory pyramidal neurons, the high density of voltage-gated sodium channels (NaV1.6) at the distal AIS regulates AP initiation. A surrogate AIS marker, ankyrin-G (ankG) is a structural protein regulating neuronal functional via clustering voltage-gated ion channels. In neuronal circuits, changes in presynaptic input can alter postsynaptic output via AIS structural-functional plasticity. Recently, we showed experimental mild traumatic brain injury (mTBI) evokes neocortical circuit disruption via diffuse axonal injury (DAI) of excitatory and inhibitory neuronal systems. A key finding was that mTBI-induced neocortical electrophysiological changes involved non-DAI/ intact excitatory pyramidal neurons consistent with AIS-specific alterations. In the current study we employed Thy1-yellow fluorescent protein (YFP)-H mice to test if mTBI induces AIS structural and/or functional plasticity within intact pyramidal neurons 2 days after mTBI. We used confocal microscopy to assess intact YFP+ pyramidal neurons in layer 5 of primary somatosensory barrel field (S1BF), whose axons were continuous from the soma of origin to the subcortical white matter (SCWM). YFP+ axonal traces were superimposed on ankG and NaV1.6 immunofluorescent profiles to determine AIS position and length. We found that while mTBI had no effect on ankG start position, the length significantly decreased from the distal end, consistent with the site of AP initiation at the AIS. However, NaV1.6 structure did not change after mTBI, suggesting uncoupling from ankG. Parallel quantitative analysis of presynaptic inhibitory terminals along the postsynaptic perisomatic domain of these same intact YFP+ excitatory pyramidal neurons revealed a significant decrease in GABAergic bouton density. Also within this non-DAI population, patch-clamp recordings of intact YFP+ pyramidal neurons showed AP acceleration decreased 2 days post-mTBI, consistent with AIS functional plasticity. Simulations of realistic pyramidal neuron computational models using experimentally determined AIS lengths showed a subtle decrease is NaV1.6 density is sufficient to attenuate AP acceleration. Collectively, these findings highlight the complexity of mTBI-induced neocortical circuit disruption, involving changes in extrinsic/presynaptic inhibitory perisomatic input interfaced with intrinsic/postsynaptic intact excitatory neuron AIS output.

## Introduction

Traumatic brain injury (TBI) constitutes a major global healthcare problem that extracts a devastating personal and societal toll (Langlois et al., 2006). Historically, TBI research focused on severe head injuries associated with high-speed motor vehicle incidents. In the last decade, the incidence of severe TBI decreased dramatically (Thurman et al., 1999; Coronado et al., 2011), with milder forms of TBI elicited by contact sports and blast waves during combat now predominating (Borg et al., 2004; Cassidy et al., 2004; Styrke et al., 2007; Hoge et al., 2008). Mild TBI (mTBI) is frequently termed the “silent epidemic” because of its relatively subtle nature typically occurring without any evidence of macroscopic brain damage such as contusion or hematoma formation (Alexander, 1995; Povlishock and Katz, 2005). However, recent findings in patients with mTBI linking acute cognitive network dysfunction with potential life-long morbidity have captured the attention of media and raised public concern (Mannix et al., 2016). Despite the focus of research shifting to mTBI (CDC, 2003), its cellular and physiological substrates remain controversial (Shaw, 2002; Büki and Povlishock, 2006; Cohen et al., 2007; Andriessen et al., 2010; Johnson et al., 2013).

Using advanced structural neuroimaging of patients with TBI ranging from mild-to-severe, multiple groups have demonstrated that diffuse axonal injury (DAI) occurs within various subcortical white matter (SCWM) tracts and callosal domains (Salmond et al., 2006; Bazarian et al., 2007; Mayer et al., 2010; Kinnunen et al., 2011). These findings led to the inference that white matter DAI causes cortical network dysfunction that underlies acute and/or chronic cognitive impairment (Bonnelle et al., 2011; Sharp et al., 2011, 2014; Johnson et al., 2013). However, other groups using the same imaging tools have not confirmed this finding in well-controlled studies of patients with mTBI and clinically significant cognitive dysfunction (Zhang et al., 2010; Mac Donald et al., 2011; Ilvesmäki et al., 2014; Wäljas et al., 2014). In fact, some reports suggest the potential for primary neocortical gray involvement (Newcombe et al., 2011; Bouix et al., 2013; Ling et al., 2013), wherein local information processing relies on balanced excitatory and inhibitory unitary neuronal activity to support function of large-scale distributed neurocognitive networks (Mountcastle, 1979; Mesulam, 1990; Douglas et al., 1995; Varela et al., 2001; Hasenstaub et al., 2005; Raichle, 2010). Several multi-modal studies using functional MRI and electrophysiological approaches have reported local neocortical network dysfunction following mTBI, supporting this premise (Mayer et al., 2011; Sponheim et al., 2011; Tremblay et al., 2011; Bashir et al., 2012; Huang et al., 2017; Palacios et al., 2017).

Although these issues are extremely difficult to rigorously assess in humans, our laboratory critically evaluated the potential for mTBI-induced neocortical change in a well-controlled animal model. Specifically, following experimental mTBI using transgenic mice, we observed primary neocortical damage involving scattered DAI within a subset of layer 5 pyramidal neurons expressing a yellow variant of green fluorescent protein (YFP) labeling the soma as well as their dendrites and axons (Greer et al., 2011). Perhaps even more biologically significant was our parallel observation that the non-DAI (intact) YFP+ neuronal population showed altered electrophysiological properties without any evidence of overt structural damage (Greer et al., 2012; Hånell et al., 2015a; Sun and Jacobs, 2016). Over time, these same intact YFP+ pyramidal neurons became hyperexcitable, entirely consistent with some form of mTBI-induced circuit disruption within local neocortical networks (Zhang and Raichle, 2010; Wolf and Koch, 2016).

At present, the neurobiological basis for this hyperexcitability in this intact neuronal population remains unknown. While several different mechanisms can alter neuronal excitability, our observations are most consistent with structural-functional changes within the axon initial segment (AIS), the site of action potential (AP) generation (Stuart et al., 1997; Palmer and Stuart, 2006). The AIS is a critical subdomain regulating neuronal excitability by integrating synaptic input to determine whether to fire an AP, thereby acting as a gatekeeper of neuronal output (Bender and Trussell, 2012; Kole and Stuart, 2012). Specifically, the high density of voltage-gated sodium channels (NaV) at the distal AIS sets the threshold for AP generation (Kole et al., 2008; Hu et al., 2009; Popovic et al., 2011). Ankyrin-G (ankG), a widely used AIS marker, is the master structural organizer that clusters ion channels via linkage to the subaxolemmal cytoskeleton (Zhou et al., 1998; Jenkins and Bennett, 2001; Yang et al., 2007). In sensory circuits, presynaptic input fine-tunes AIS structure (Kuba, 2012; Gutzmann et al., 2014; Kuba et al., 2014) altering neuronal excitability (Kuba et al., 2010). Because pyramidal neuron AIS function is strongly regulated by perisomatic GABAergic synaptic transmission from parvalbumin (PV)-expressing interneurons (Cobb et al., 1995; Pouille and Scanziani, 2001; Freund, 2003; Klausberger et al., 2003; Klausberger and Somogyi, 2008; Cardin et al., 2009; Atallah et al., 2012; Pouille et al., 2013; Lazarus et al., 2015; Wefelmeyer et al., 2015), concomitant involvement of these inhibitory inputs could potentially induce changes in AIS structure-function after mTBI (Buffington and Rasband, 2011; Baalman et al., 2013).

To address these AIS-related issues, the current report builds upon our previous studies that utilized YFP-expressing transgenic mice in a well-characterized model of mTBI to examine the potential for changes in layer 5 pyramidal neuron AIS and its associated perisomatic domain, both of which are critical for regulating neuronal excitability. Specifically, to assess AIS structural plasticity, immunohistochemical approaches targeting ankG and NaV1.6 distribution were quantitatively assessed in the early stage post-mTBI using confocal microscopy. These assessments of AIS structural plasticity in non-DAI/intact layer 5 pyramidal neurons were also accompanied by parallel morphological studies examining presynaptic GABAergic bouton density. Lastly, these imaging studies were interfaced with patch-clamp electrophysiology and computational modeling of layer 5 pyramidal neurons to establish a functional match with the AIS structural plasticity posited above. Using these multifaceted approaches, we show for the first time that mTBI evokes structural plasticity at the distal AIS, occurring independent of detectable changes in sodium channel distribution, in a specific subset of neocortical neurons with intact axons. These same events within intact neurons were also recognized to occur in concert with PV+ GABAergic terminal loss at their perisomatic domain. Lastly, the observed intrinsic/postsynaptic (AIS) and extrinsic/presynaptic (perisomatic) structural plasticity within intact neurons were also accompanied by a reduction in AP acceleration, likely reflecting a change in NaV1.6 density. Collectively, these findings reshape our understanding of mTBI and have major implications as we continue to better understand the brain’s response to milder injury in terms of the functionality of its intrinsic networks within neocortex.

## Materials and Methods

Animal maintenance and experimental protocols were approved by the Virginia Commonwealth University Institutional Animal Care and Use Committee and complied with principles in the National Research Council *Guide for the Care and Use of Laboratory Animals: 8th Edition*.

### Breeding and Genotyping of YFP-H Transgenic Mice

The Thy1-YFP-H line [B6Cg-TgN(Thy1-YFP-H)2Jrs, stock number 003782] was obtained from Jackson Laboratories (Bar Harbor, ME) and maintained as heterozygotes upon a C57BL/6J background. Inheritance of the fluorescent transgene in these was determined from an ear punch taken at weaning (~21 days). The tissue from the ear punch was mounted on a glass slide and examined using a FITC filter on an Olympus DP71 digital camera (Olympus, Center Valley, PA, USA) where YFP expression could easily be identified in mice that inherited the transgene (YFP-H mice). Transgene expression in these mice is under the control of the neuronal specific Thy1 promoter, resulting in YFP expression within the neocortex that is primarily restricted to layer 5 pyramidal neurons (Feng et al., 2000).

### Experimental Design

This study used a total of 18 young adult (age: 6–10 weeks; weight: 20–24 g) male mice randomly assigned^1^ to either sham-injury (control) or mTBI (experimental) groups. Based on our previous work showing mTBI consistently generates DAI within primary somatosensory barrel field (S1BF), we assessed layer 5 intact YFP+ pyramidal neurons for AIS structural-functional changes at 2 days postinjury within this well-characterized region of neocortical gray matter (Greer et al., 2011, 2013; Hånell et al., 2015b). For confocal microscopy studies, a total of 11 mice (age 8–10 weeks) were surgically prepared for sham-injury (*n* = 5) or mTBI (*n* = 6) and perfused for immunohistochemical labeling of tissue sections. To identify the AIS we fluorescently labeled ankG, the master scaffolding protein that is widely used as a surrogate marker for AIS position and length (Hedstrom et al., 2008; Grubb and Burrone, 2010; Gutzmann et al., 2014). Additionally, we targeted NaV1.6, which sets the threshold for AP generation (Kole et al., 2008), to structurally assess the membrane channels underpinning AIS function/output. In addition to intrinsic/postsynaptic AIS structural plasticity within intact YFP+ pyramidal neurons, we assessed their perisomatic domain for extrinsic/presynaptic changes in GABAergic bouton density. To probe for any AIS functional plasticity correlated with potential structural changes we recorded intrinsic electrophysiological data from intact YFP+ pyramidal neurons in layer 5 of S1BF within *ex vivo* slice preparations from a total of 7 mice (age 6–8 weeks) at 2 days following sham-injury (*n* = 3) or mTBI (*n* = 4). Lastly, we determined the functional consequences of any potential AIS structural changes using a realistic computational model of a layer 5 pyramidal neuron based on experimentally derived properties (Hallermann et al., 2012).

### Surgical Preparation and Central Fluid Percussion Injury

To model mTBI we used midline central fluid percussion injury (cFPI) first described by Dixon and associates using rats (Dixon et al., 1987), which our lab modified for mice as described previously (Greer et al., 2011). Briefly, anesthetized mice were surgically prepared for cFPI induction by installing a hub surrounding a craniectomy centered on the superior sagittal suture, midway between bregma and lambda. Intraoperative rectal temperature was maintained at 37 ± 0.2°C using a thermostatically controlled heating pad (Harvard Apparatus). Additionally, heart rate beats per minute (BPM), respiratory rate (RPM) and arterial blood oxygenation (SpO_2_) were monitored using a thigh-clamp pulse oximeter sensor (MouseOx; STARR Life Sciences) to ensure maintenance of physiological homeostasis. After a post-operative recovery (~1.5 h), mice were re-anesthetized and then connected to the fluid percussion apparatus (Custom Design and Fabrication, Virginia Commonwealth University) forming a closed mechanical system. Releasing the pendulum, striking the piston in the fluid-filled cylinder generated a mild pressure wave (~12 ms) that was delivered onto the intact dura. This action simulates human brain inertial loading during trauma-induced rapid acceleration-deceleration causing a mild diffuse brain injury (Dixon et al., 1987). The mean ± standard error of the mean (SEM) of the peak amplitude of the pressure wave (1.6 ± 0.03 atmospheres, *n* = 10 mice) was measured by a transducer and displayed on an oscilloscope (Tektronix TDS 210). For sham-injury, an identical procedure was used with the exception of the pendulum’s release. Mice were disconnected from the apparatus immediately postinjury and visually monitored while removing the hub, suturing the incision and checking reflexes. None of the mice showed signs of seizure or apnea. Severity of injury (peak amplitude) and duration of loss of righting reflex, a rodent behavioral surrogate of loss of consciousness, were recorded for each animal (Grimm et al., 2015). We determined the degree of mTBI by comparing loss of righting reflex duration with shams (Morehead et al., 1994). After recovering from loss of righting reflex, animals were transferred to a warmed cage to maintain normothermia and monitored before returned to the vivarium.

### Perfusion and Tissue Processing

Mice received a lethal dose of sodium pentobarbital (1.6 mg/g IP) 2 days postinjury. After loss of pain reflexes mice were transcardially perfused, first with heparinized (10 units/ml) saline for 1 min then 4% paraformaldehyde in Millonig’s buffer pH 7.4 for 20 min. Brains were dissected and then sectioned coronally at 60 μm using a vibratome (Leica VT1000S). Sections directly below the craniectomy (bregma level −0.6 to −2.4 mm) were collected in 24-well plates filled with Millonig’s buffer pH 7.4. To quantitatively assess for intrinsic AIS and extrinsic/synaptic perisomatic structural plasticity, for each animal, we labeled ankG, NaV1.6 and double-labeled glutamate decarboxylase-67 (GAD67) with PV in sections taken from a randomly selected and two adjacent wells, respectively, containing caudal S1BF (bregma level −1.5 to −2.0). This was done because of the consistency with which cFPI generates DAI within this well characterized area of neocortex, which was also the rational for choosing this region-of-interest (ROI) in our previously published electrophysiological studies (Greer et al., 2012; Hånell et al., 2015a; Sun and Jacobs, 2016).

### Immunohistochemistry

Free-floating sections were rinsed with phosphate buffered saline (PBS). Heat-induced epitope retrieval was performed by incubating sections in 10 mM sodium citrate buffer pH 8.5 for 10 min in an 80°C water bath (Jiao et al., 1999). After cooling to room temperature sections were rinsed with PBS then incubated for 1 h at room temperature with 10% normal goat serum, 2% fish skin gelatin and 0.5% Triton X-100 in PBS. To mask any potential endogenous mouse immunoglobulin, the blocking buffer was supplemented with Mouse-on-Mouse reagent (Vector Laboratories, MKB-2213). Then, sections were rinsed with 1% normal goat serum, 1% fish skin gelatin, and 0.5% Triton X-100 in PBS (working buffer). Primary antibody solutions were prepared by dilution with working buffer and the sections were incubated overnight at 4°C with agitation. Specifically, we used monoclonal antibodies against ankG (1:500; mouse IgG2a, clone N106/36; NeuroMab), NaV1.6 (1:200; mouse IgG1, clone K87A/10; NeuroMab), PV (1:2000; mouse IgG1; Swant, PV235) and GAD67 (1:1000; mouse IgG2a, clone 1G10.2; Millipore, MAB5406). For qualitative double-labeled overview images, we used a polyclonal antibody against NaV1.6 (1:200; Alomone). The following day, sections were rinsed with working buffer and incubated with isotype-specific goat-derived secondary antibodies conjugated to Alexa Fluor 568 for quantitative analyses and a combination of Alexa Fluor 568 and 633 for qualitative colocalization (1:500: ThermoFisher Scientific) for 2 h at room temperature. After final rinses using working buffer then PBS, sections were mounted on glass slides and cover-slipped using non-hardening Vectashield with DAPI (Vector Laboratories, H-1200) to prevent shrinkage and preserve morphology of tissue.

The AIS quantitative analysis on the YFP+ background used separate single-labeled sections opposed to double-labeled single sections in an effort to match the fluorescent quantum yield and stoichiometry via monoclonal primary antibodies to immunolabel ankG and NaV1.6, which were both visualized using Alexa Fluor 568 (Lichtman and Conchello, 2005). In the same vein, mouse immunoglobulin isotype-specific secondary antibodies were used in all studies, which also optimized the signal-to-noise ratio (Manning et al., 2012). Parallel control studies were conducted to ensure both primary and secondary antibody fidelity (Lorincz and Nusser, 2008b). In all cases, primary antibody omission abolished immunoreactivity. Secondary antibody specificity evaluated via cross-reactivity showed no signal between all possible primary × secondary host and/or isotype combinations.

### Confocal Microscopy

Image acquisition was performed using a laser-scanning confocal microscope (LSM 710, Carl Zeiss). Since sham vs. mTBI groups could be readily differentiated based on YFP+ profiles, we used DAPI visualized under epifluorescence to center the stage over the ROI. In this way, the investigator was blinded from the experimental/dependent variable channel, thus adhering to stereological principles including random sampling. Using a 10× objective (low-power) the field-of-view (FOV) was centered over the S1BF region along the dorsolateral edge of the hippocampus. We used continuous laser scanning to guide rotation of the FOV until it was orthogonal to the underlying SCWM. Images were acquired with optimal Nyquist sampling using Plan-Apochromat 10×/0.45 NA (*XY* = 0.41 μm/pixel; *Z* = 5.8 μm), 20×/0.8 NA (*XY* = 0.152 μm/pixel; *Z* = 0.94 μm), 40×/1.4 (*XY* = 0.094 μm/pixel; *Z* = 0.52 μm) and 63×/1.2 NA (XY = 0.088 μm/pixel) oil immersion objective lenses. All multichannel images were acquired using sequential scanning at the lowest possible laser power to avoid crosstalk (488 Argon, 561 DPSS and 633 HeNe). All images for quantitative analysis were acquired using identical laser settings per channel across samples. Gain and offset were adjusted for optimal signal range. The pinhole was set to 1.0 Airy unit for the red channel (i.e., ankG, NaV1.6 and GAD67). To maintain identical optical slice thickness with respect to the red channel, in multichannel *z-stack* images the green (YFP) and far-red (PV) the pinhole ranged from 0.8 to 1.2 Airy units.

### Intact YFP+ Pyramidal Neuron Sampling

Within the 10× overview images we measured ever YFP+ pyramidal neuron that met our inclusion criteria. Specifically, intact neurons were defined by YFP+ axons that were continuous from the soma of origin to the SCWM interface. YFP+ that were transected during tissue processing were excluded in both sham and mTBI samples. Since AIS structure and composition vary even within a particular neuronal subtype (Kuba, 2012; King et al., 2014), we set *a priori* conditions to control for any potential confounds. Specifically, layer 5 is subdivided into layer 5a and 5b, which are populated by multiple subtypes of pyramidal neurons with different morphological and physiological properties, as well as projections to different anatomical regions of the brain (Chagnac-Amitai et al., 1990; Schubert et al., 2006; Hattox and Nelson, 2007). To control for potential confounding of layer 5 pyramidal neurons based on neocortical depth, for each AIS sample we also measured the distance from the axo-somatic vertex to where YFP+ profiles entered SCWM, indicated by the sharp change in trajectory. Additionally, we excluded pyramidal neurons with axons emerging from basal dendrites because of their unique intrinsic and synaptic properties (Thome et al., 2014; Hamada et al., 2016). Overall, the number of intact YFP+ pyramidal neurons meeting our inclusion criteria ranged from 5 to 14 per section. This data set met the recommended minimum of five measurements per animal to obtain a robust and unbiased estimates of variance using multilevel modeling described below (Walsh, 1947; Maas and Hox, 2004; Galbraith et al., 2010). Further, the Wilcoxon test showed sample sizes between sham and mTBI groups were similar for both ankG (sham: *n* = 53 AIS from 5 mice; mTBI: *n* = 54 AIS from 6 mice; *X*^2^ = 0.079, *p* = 0.7782) and NaV1.6 (sham: *n* = 54 AIS from 5 mice; mTBI: *n* = 44 AIS from 6 mice; *X*^2^ = 3.36, *p* = 0.0666) analyses of AIS structure. These samples sizes allowed us to detect a 1.5 μm (5%–10%) change in AIS end position with 80% power (G^*^Power; Franz Faul, Kiel University) based on mean ± SD sham ankG end position (25.5 ± 2.94 μm; *n* = 50 AIS from 3 mice) determined from pilot experiments. For perisomatic GABAergic bouton density, sample sizes between sham (*n* = 48 somas from 5 mice) and mTBI (*n* = 50 somas from 5 mice; one section lost during processing) groups were also similar (*X*^2^ = 0.1, *p* = 0.7518; Wilcoxon test).

### Quantification of AIS

To capture ankG and NaV1.6 fluorescent profiles along YFP+ intact pyramidal neurons, a 40× objective at 3× zoom (760 × 760 pixels) in *z*-stacks (mean 4.5 μm, range 2.7–5.3 μm). Because of the diffuse nature of YFP+ pathology following mTBI, these constrained images allowed tracing the YFP+ axon through and past the AIS region in a blinded fashion. Further, we set a limit of 10 optical slices to minimize error caused by tilted AIS orientation in the *z*-plane. AIS measurements were performed using a previously described method (Grubb and Burrone, 2010; Evans et al., 2013). Specifically, *z-stacks* were collapsed into a single maximum intensity projections that were imported into MATLAB software (MathWorks) for analyses using custom-written functions (Matthew Grubb and Thomas Watkins, King’s College London, UK; freely available at www.mathworks.com/matlabcentral/fileexchange/28181-ais-quantification). While visualizing only the YFP channel, an axonal profile was traced starting at the edge of the soma, continuing distally along the axon, through and past the region of the AIS. Immunofluorescent profiles of ankG/NaV1.6 were then superimposed on traces of YFP+ axons. At each pixel along this profile, fluorescent intensity values were averaged over a 3 × 3 pixel square centered on the pixel of interest. Averaged profile values were then smoothed using a 40-point (~5 mm) sliding mean and normalized between 1 (maximum smoothed fluorescence) and 0 (minimum smoothed fluorescence). With respect to the soma edge, delineated at the axo-somatic vertex, ankG and NaV1.6 proximal (start) and distal (end) positions were obtained at points where the profiles reached thresholds of 0.33 and 0.5 relative to maximum fluorescence, respectively. These optimal threshold values were determined empirically and varying ± 0.2 did not change the overall pattern of results.

### Quantification of Perisomatic Bouton Density

The total number of GAD67+ and GAD67+/PV+ puncta were quantified along the perisomatic domain of YFP+ intact pyramidal neurons within layer 5 of S1BF. Single optical slices (0.9 μm thick) were captured using a 63× objective at 3× zoom (512 × 512 pixels, 45 μm × 45 μm; resolution = 0.088 μm/pixel) using sequential scanning and a single laser intensity for each channel for all samples. Confocal images were imported to Fiji (a distribution of ImageJ) then processed and analyzed using custom written macros for automated analyses. YFP images were converted into binary images to segment the pyramidal neuron profile, which was then dilated and eroded to generate a 2 μm thick band approximating the perimeter/perisomatic domain. Quantification of puncta within this band was performed using both gray scale and segmented/binary images. Specifically, perisomatic GAD67+ puncta in gray scale (8-bit) images were quantified using the “Find Maxima” function. To quantify perisomatic GAD67+/PV+ puncta, profiles in each channel were segmented from background subtracted 8-bit images by converting into binary using a minimum gray-value threshold. Varying the threshold ±10 gray-values did not change the overall pattern of results. GAD67+/PV+ colocalized populations were isolated using the “Image Calculator” function. The “Particle Analysis” function with appropriate size and shape exclusion filters was used to determine the total number of objects per unit area (FOV). GAD67+ (PV+) puncta within this band were quantified and normalized by dividing by the area of the band to determine perisomatic bouton density, summarized as puncta per 100 μm^2^.

### Computational Modeling

Simulations in Neuron v7.1 used a previously published realistic model of neocortical layer 5 pyramidal neuron AP initiation (Hallermann et al., 2012). The model was based on Neurolucida reconstructions using ion channel properties determined from experimental recordings. Simulations were executed using default setting with the exception of AIS ion channel densities and length modifications based on our experimental observations. Specifically, the default AIS length (63 μm) in this model was ~60% longer than our experimental measurements. To compare the effect of AIS length using our experimentally observed values (~24–26 μm), the peak NaV1.6 density at the distal AIS was increased proportionally from the default value of 7000–11,200 pS μm^−2^. Importantly, this control NaV1.6 density remained within the realistic estimates reported by Hallermann et al. (2012). Default current-clamp parameters were used to evoke an AP within 5 ms of injection. Overall, simulations using our adjusted AIS parameters resulted in AP waveforms that were qualitatively similar to those produced using default settings and also our electrophysiological recordings. This allowed us to investigate the kinetics (i.e., acceleration; described below) of the AP at the AIS in isolation from the AP at the soma. Specifically, we compared the effect of changing AIS length using experimentally determined values (i.e., sham vs. mTBI) and NaV1.6 density on AP acceleration. Note, in these layer 5 pyramidal neuron simulations, AIS length was modulated at the distal position with respect to the soma of origin. Additionally, changing length or peak NaV1.6 density at the distal AIS did not change the distribution ratio of ion channels.

### Electrophysiology

In addition to the above, a separate cohort of sham-injured (*n* = 3) and mTBI (*n* = 4) mice were anesthetized with isoflurane and decapitated for quick brain removal 2 days postinjury. The brains were immediately chilled in ice-cold oxygenated sucrose-modified artificial cerebral spinal fluid (aCSF) slicing solution (mM: 2.5 KCl, 10 MgSO_4_, 0.5 CaCl_2_, 1.25 NaH_2_PO_4_, 234 sucrose, 11 glucose and 26 NaHCO_3_). Using a vibratome (VT 1200, Leica Microsystems) brains were coronally sectioned at 300 μm and then incubated for 30–45 min at 34°C in an oxygenated aCSF (mM: 126 NaCl, 3.5 KCl, 1 MgSO_4_, 1.2 CaCl_2_, 1.25 NaH_2_PO_4_, 10 glucose and 26 NaHCO_3_). Thereafter, slices remained at room temperature until placed in the recording chamber maintained at 32 ± 0.5°C.

As previously described (Sun and Jacobs, 2016), whole-cell patch-clamp recordings were performed under infrared Dodt contrast microscopy (Zeiss AxioExaminer). A 60× water-immersion objective was used to visually identify YFP+ layer 5 pyramidal neurons of S1BF with axons descending from the soma into the white matter (intact) or ending with an axonal swelling (DAI), deep to the surface of the slice to avoid those transected by the vibratome. We have shown previously that these morphologies are easily identified in the living slice for YFP+ layer 5 pyramidal neurons (Greer et al., 2012). Consistent with our structural assessments, only these intact YFP+ pyramidal neurons were recorded in both sham and mTBI slices. Additionally, all layer 5 YFP+ samples had an apical dendrite, a characteristic morphological feature of pyramidal neurons. The slices were continuously perfused with aCSF solution saturated with 95% O_2_ and 5% CO_2_. Patch electrodes (final resistances, 2–4 MΩ) were pulled from borosilicate glass (World Precision Instruments) on a horizontal Flaming-Brown microelectrode puller (Model P-97, Sutter Instruments). The intracellular solution contained (in mM): 130 K-gluconate, 10 Hepes, 11 EGTA, 2.0 MgCl_2_, 2.0 CaCl_2_, 4 Na-ATP and 0.2 Na-GTP. Electrode capacitance was electronically compensated. Data were acquired and digitized at 200 kHz using a MultiClamp 700B amplifier and Digidata 1440A with pClamp software, respectively (Molecular Devices). Whole-cell patch-clamp was approached under voltage-clamp mode at −65 mV. After 2–5 min stabilization, APs were recorded in current-clamp mode to obtain intrinsic property measurements. Pipette Capacitance Neutralization was set at 9.6 pF, Bessel Filter at bypass, auto Bridge Balance, and Gain at one. APs were evoked with 10 depolarizing steps (30 ms) beginning with a 100 pA step, and increased by 10 pA every 5 s while neurons were maintained at −60 mV. Access resistance was continuously monitored and rechecked after each recording. If the series resistance increased by 20% at any time, the recording was terminated. Previously we showed that there was no significant difference between the results for naïve and sham animals (Greer et al., 2012). Despite this similarity, for added rigor the control group contained only age- and survival time-matched sham-injured animals. Additionally, passive membrane properties (mean ± standard deviation; SD) compared using a *t* test showed no statistical differences in capacitance (pF: sham = 196 ± 46.5, mTBI = 176 ± 28.4; *t*_25_ = −1.35, *p* = 0.1882), input resistance (MΩ: sham = 67 ± 21.5, mTBI = 79 ± 25.1; *t*_25_ = 1.28, *p* = 0.2111), access resistance (MΩ: sham = 9.2 ± 3.21, mTBI = 9.11 ± 3.06; *t*_25_= −0.07, *p* = 0.9409), or resting membrane potential (mV: sham = −69.8 ± 3.03, mTBI = −70.7 ± 5.79; *t*_25_ = −0.49, *p* = 0.6298) between sham (*n* = 13 cells from 3 mice) and mTBI (*n* = 14 cells from 4 mice) groups.

In this report, we use a newly implemented protocol for intrinsic property analysis that allows us to measure the first AP on each sweep (5 sweeps per cell). To assess AP kinetics, we applied three successive seven-point boxcar filters to the membrane voltage, and then calculated the first and then second derivative, which yielded two peaks. Specifically, the first and second peak in the plot of the second derivative of the membrane voltage corresponded to AP acceleration at the AIS and soma, respectively (Khaliq and Raman, 2006; Meeks and Mennerick, 2007). All recorded cells from sham and mTBI slices resulted in two peaks, and their amplitudes were measured for each of five APs per cell. For statistical analyses, the first AP on each of the five sweeps was nested within each cell. There were two cells in sham slices where only four sweeps were recorded. In total, we recorded and analyzed 63 AP from sham and 70 AP from mTBI mice. The number of recorded cells per animal, (range = 2–6) was similar (*X*^2^ = 0.5283, *p* = 0.4673) between sham (*n* = 3 mice) and mTBI (*n* = 4 mice). For statistical analysis using multilevel modeling described below, each neuron had and additional nested set of five APs.

### Statistics

Statistical analysis of data sets was performed using JMP Pro version 12.2.0 (a distribution of SAS). Data sets were assessed for normality using quantile (QQ) plots and the Shapiro-Wilk test. For all analyses, the variance in data between sham and mTBI groups was not significantly different (*p* > 0.05, Brown-Forsythe test). Physiology data and loss of righting reflex were statistically analyzed using unpaired *t* tests. Statistical analyses (described below) tested for covariance of distance from SCWM with AIS measurements. If covariance was significant, AIS measurements were leveraged with respect to distance from SCWM. Non-leveraged vs. leveraged AIS measurements were statistically assessed for significant difference using the Wilcoxon signed-rank test. For this test, the statistical unit corresponded to the average difference (non-leveraged−leveraged) in AIS measurements per animal.

To determine statistically significant differences among intact YFP+ pyramidal neuron outcome variables (distance from SCWM, AIS structure, perisomatic bouton density and AIS function) between sham-injury and mTBI groups, we employed multilevel modeling (Nieuwenhuis et al., 2011; Button et al., 2013; Aarts et al., 2014). For statistical analysis, we constructed a multilevel model using an experimental condition at the animal level (sham-injury or mTBI), where the outcome variable (e.g., AIS length) consists of multiple measurements per animal (i.e., nested data). In other words, each animal had nested data consisting of a series of measurements. Because some variability is expected in the average outcome measure as a function of each animal, the data from each animal are not independent (Nieuwenhuis et al., 2011). Thus, failure to account for such dependent data can inflate the Type I error rate (α) above the standard nominal value of 0.05 (Snijders and Bosker, 1993). Multilevel modeling accounts for this within-animal dependence on the outcome measure (e.g., AIS length) and thus preserves the true Type I error rate for the statistical test (Walsh, 1947; Galbraith et al., 2010), which we have set to *α* = 0.05.

Statistical analysis using multilevel modeling performs linear regressions in a sequential fashion (Aarts et al., 2014). In addition to accounting for intra-animal variability, this statistical model also allowed us to test whether distance from SCWM was a covariate of AIS length, and if there were any interactions with sham vs. mTBI groups. To conduct multilevel modeling, first an *F* test was used to determine if the multilevel model accounted for the variability in the data set. If the *F* test yielded *p* < 0.05, we inferred that the model fit the data and continued our analysis. We then evaluated the adjusted coefficient of determination (*R*^2^), which describes how much of the variability in the data is accounted for in the multilevel model (e.g., the proportion of AIS length that is a function of mTBI and distance to SCWM). In predictive statistics, the *R*^2^ can be used to assess the effect size of a multilevel linear regression model, where 0.01, 0.09 and 0.25 are defined as small, medium and large effects, respectively (Aarts et al., 2014). In next step, a two-tailed *t* test in linear regression terms was used to determine whether each specific parameter (e.g., sham vs. mTBI and distance to SCWM) had a significant effect on the outcome measure (e.g., AIS length), with the threshold for significance set at *α* < 0.05 adjusted for multiple comparisons. When analyzing only two experimental groups (e.g., sham vs. mTBI), a generally accepted index for reporting a standardized effect size is Cohen’s *d*, which equals the difference in the means of the two groups in units of SD. Cohen defined 0.2, 0.5 and 0.8 as small, medium and large effects, respectively (Cohen, 1988).

For ankG multilevel modeling, intact YFP+ pyramidal neuron summary statistics (*t* ratio, degrees of freedom, and *p*-value) and outcome measures (mean ± SEM) were calculated from nested data sets (e.g., *N* = 53 AIS total, 10–11 AIS nested per animal, *n* = 5 mice). To summarize multilevel modeling statistics, we report the number of animals per group (*n*) and total number of measurements per animal (*N*), the degrees of freedom (denoted as the subscript in the *t* ratio, and the *p*-value). All statistical data are summarized using mean ± SEM, unless otherwise noted. Descriptive data are summarized with mean and 95% confidence intervals (CI). All statistical tests were two-tailed and considered significantly different for *p* < 0.05.

## Results

Intraoperative physiology was normal and consistent with previous reports on mice under isoflurane anesthesia (Cesarovic et al., 2010; Ewald et al., 2011; Hånell et al., 2015b). Specifically, the arterial oxygen saturation (sham = 97.8 ± 0.1%; mTBI = 97.7 ± 0.2%), heart rate (sham = 513 ± 12 BPM; mTBI = 505 ± 14 BPM) and RPM (sham = 63 ± 6 RPM; mTBI = 74 ± 6 BRPM) were similar between sham (*n* = 8 mice) and mTBI (*n* = 10 mice) groups (SpO_2_: *t*_12.8_ = 0.73, *p* = 0.4770; BPM:* t*_16.0_ = 0.42, *p* = 0.6811; RPM: *t*_15.4_ = −1.23, *p* = 0.2251; *t* test). Consistent with our previous reports (Greer et al., 2011; Hånell et al., 2015b), the loss of righting reflex duration in mTBI mice (4.7 ± 0.24 min; *n* = 10) was significantly greater (*t*_11.4_ = 7.13, *p* < 0.0001; *t* test) than shams (1.2 ± 0.42 min; *n* = 8). Additionally, mTBI mice for confocal (4.7 ± 0.39 min; *n* = 6) and electrophysiological (4.6 ± 0.25 min; *n* = 4) studies had similar loss of righting reflex (*t*_7.8_ = −0.35, *p* = 0.7337; *t* test). Importantly, signs of hypoxia/apnea were not observed during surgical preparation or postinjury. Overall, these physiological assessments did not show any evidence of confounding mechanisms that play a role in secondary insults.

**Figure 1:**
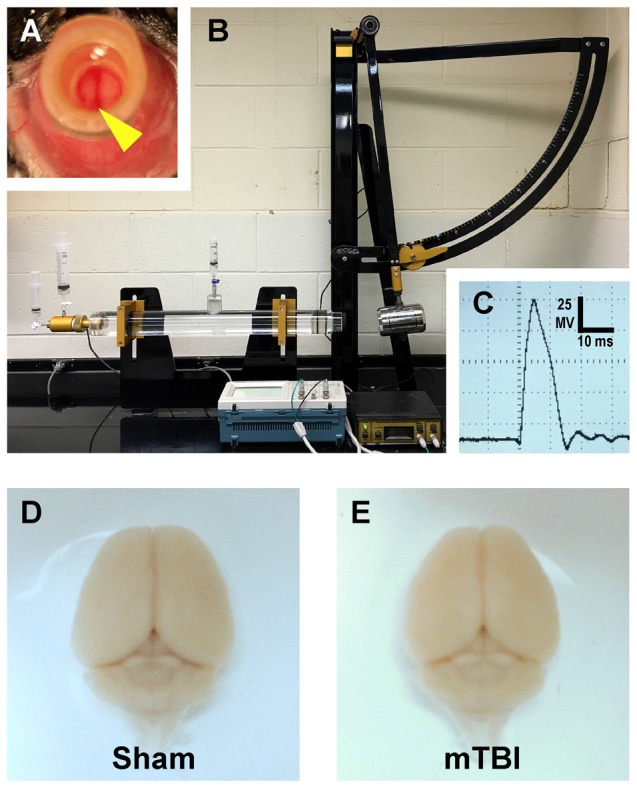
Mouse model of mild traumatic brain injury (mTBI) via midline central fluid percussion injury (cFPI). **(A)** Dorsal view showing craniectomy site with the dura intact. The superior sagittal sinus (yellow arrowhead) can be seen through the saline-filled hub. **(B)** Fluid percussion injury device with the pendulum in the cocked position. Severity of injury is determined by arc height. After releasing the trigger the pendulum strikes a piston in a cylinder reservoir generating a fluid pressure wave. The mouse cranium is attached to the cylinder at the opposite end via the installed hub, forming a closed mechanical system. **(C)** A representative fluid pressure wave that is transmitted directly onto the intact dura. **(D,E)** Representative images of dissected brains perfused 2 days after cFPI-induced mTBI. Sham **(D)** and mTBI **(E)** brains show no evidence of surgically-induced damage. After cFPI, there is no evidence of mass lesions and/or contusion. There is no focal damage at the craniectomy site where the pressure wave entered the cranium.

**Figure 2:**
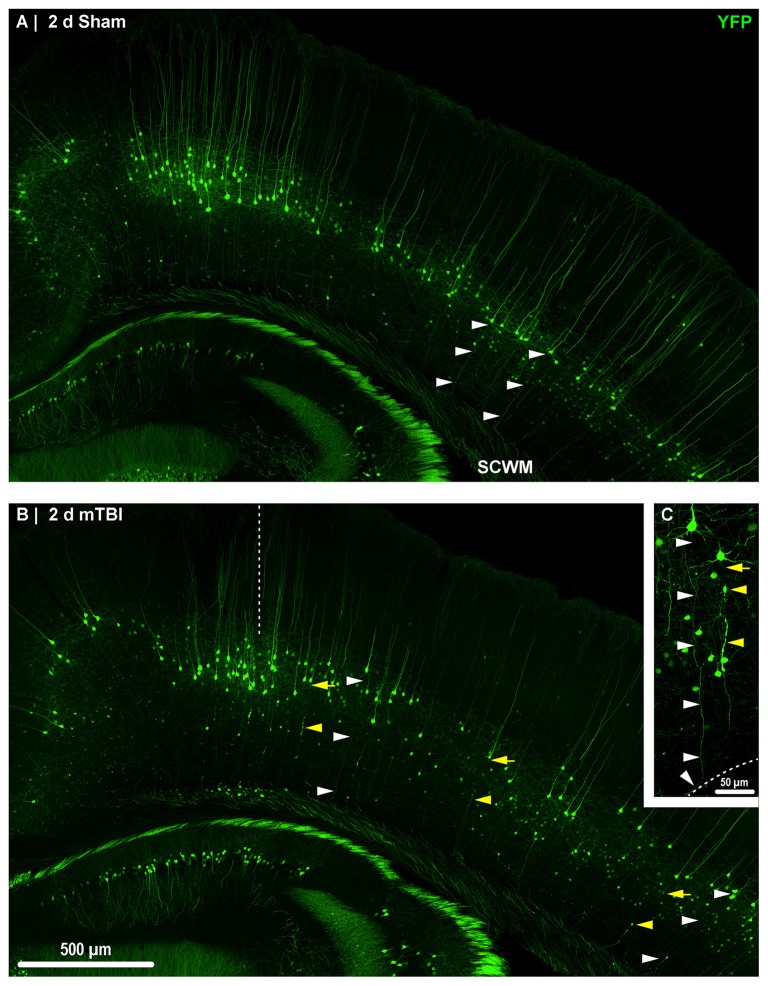
Yellow fluorescent protein (YFP) expression in neocortical layer 5 pyramidal neurons. Representative images of sections from sham **(A)** and mTBI **(B)** brains shown in Figure 1. Thy1 driven YFP expression results in restricted labeling of a discrete subset of layer 5 pyramidal neurons. **(A–C)** The selective YFP expression allows identification of individual axons that be traced from their soma of origin to the subcortical white matter (SCWM; white arrowheads). **(B)** Two days after mTBI, evidence of diffuse axonal injury (DAI) indicated by YFP+ axonal swellings is seen dispersed across the dorsolateral neocortex (yellow arrowheads). The craniectomy boundary is depicted by the vertical dashed line. No evidence of focal injury is observed under the craniectomy site. **(C)** A representative intact YFP+ pyramidal neuron juxtaposed by an injured axon. The intact axonal profile can be continuously traced from the soma of origin to where it enters the SCWM (dashed line), indicated by the acute change in trajectory.

Macroscopically, both sham and mTBI brain tissue appeared normal without evidence of surgically induced lesions (Figures 1A–E), as previously reported. Post-mTBI tissue sections revealed no macroscopic change (Figure 2) consistent with the mild and diffuse nature of cFPI. Importantly, the dorsal neocortex underneath the craniectomy site did not show evidence of focal contusion, cavitation, or overt subarachnoid hemorrhage induced by the fluid pressure wave (Figure 2B). Overall, the brain parenchyma was devoid of overt hemorrhage, the ventricular system maintained a regular contour, with no evidence of trauma-related ventricular enlargement. Taken together these data support our premise that cFPI in YFP-H mice is a reproducible model of mTBI.

### YFP+ Pyramidal Neuron DAI

**Figure 3:**
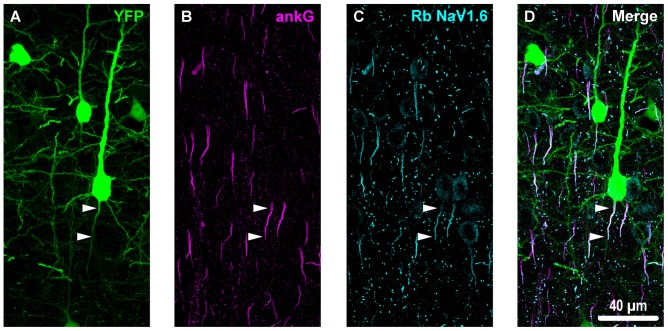
Overview of ankyrin-G (ankG) and voltagegated sodium channels (NaV1.6) immunofluorescent profiles in YFP mice. **(A)** Neocortical layer 5 YFP+ pyramidal neurons (green). **(B)** AnkG immunoreactivity visualized using Alexa Fluor 568 (magenta) shows characteristic tapered morphology. **(C)** NaV1.6 immunolabeled with a polyclonal rabbit (Rb) antibody visualized using Alexa Fluor 633 (cyan) reveals shorter profiles corresponding to the distal axon initial segment (AIS). Punctate profiles indicate Nodes of Ranvier. **(D)** Composite image clearly demonstrating the utility of ankG as an AIS marker. The proximal axon of the YFP+ pyramidal neuron in the center of the field-of-view (FOV) colocalizes with ankG and NaV1.6, which is expressed at the distal AIS. Several ankG+ profiles highlight NaV1.6 expression at the distal AIS.

**Figure 4:**
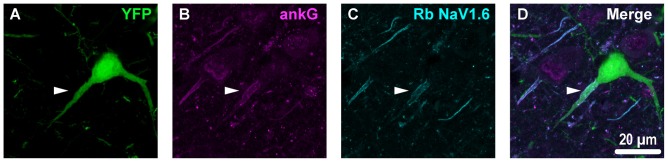
YFP+ pyramidal neuron axonal injury attenuates ankG and NaV1.6 expression at the AIS. **(A–C)** Representative images of YFP **(A)** ankG **(B)** and NaV1.6 **(C)**. **(A)** Evidence of DAI occurring at the AIS of a YFP+ pyramidal neuron (green) is indicated by a severely swollen proximal segment (arrowhead). **(B,C)** AnkG (magenta) and NaV1.6 (cyan) expression persists, but is substantially attenuated with abnormal morphology after mTBI-induced AIS injury. **(D)** Composite image showing ankG and NaV1.6 within the swollen AIS of a YFP+ pyramidal neuron.

Consistent with previous work from our laboratory and others that utilized the YFP-H strain (Feng et al., 2000; Greer et al., 2011; Antón-Fernández et al., 2015; Hånell et al., 2015b), we observed YFP+ pyramidal neurons in sham (Figures 3A,D) and mTBI (Figures 4A,D) mice primarily within layer 5 neocortex (Figure 2). Following sham-injury, we did not observe any YFP+ axonal swellings or morphological irregularities indicative of axonal damage within neocortex or underlying SCWM (Figure 2A). Descending axons from YFP+ pyramidal neurons in shams demonstrated a continuous trail from their soma of origin to the SCWM interface. Following cFPI, all tissue sections processed for YFP visualization and parallel immunohistochemical analyses revealed a pattern of microscopic change consistent with that routinely described in rat and mouse cFPI models. Despite the overall preservation of brain parenchymal integrity typical of a mild diffuse TBI in both humans and rodents, the fluid pressure wave consistently evoked YFP+ axonal swellings indicating DAI. At 2 days post-mTBI, comparable to previous reports, we observed pathologic YFP+ axonal profiles in continuity with their somas of origin (Figures 2B, 4A) and their detached axonal segments distributed throughout layer 5/6 of the dorsolateral neocortex corresponding to S1BF (Figure 2B). Underscoring the diffuse nature of mild cFPI, the distribution of YFP+ pyramidal neurons proximal and distal axonal swellings were interspersed among numerous YFP+ profiles showing no morphological evidence of either primary axonal injury or retrograde neuronal involvement. Similar to shams, we readily identified these non-DAI/intact YFP+ pyramidal neurons via tracing YFP+ axons continuously from the edge of soma of origin to the SCWM interface (Figure 2C). Importantly, we observed consistent YFP+ pyramidal neuron axonal injury within the same neocortical regions of all mTBI mice, indicating a generalized diffuse response to cFPI-induced mTBI in mice rather than an isolated focal event.

### Intact YFP+ Pyramidal Neuron Responses

#### AIS Structural Plasticity

**Figure 5:**
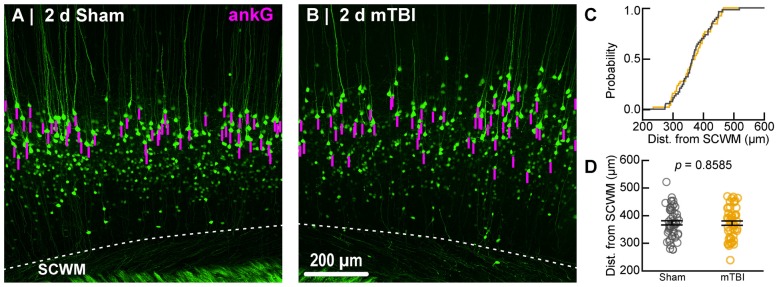
Sampling distribution of intact YFP+ pyramidal neurons for ankG quantitative analysis. **(A,B)** Superimposed maximum intensity projections of 10× overview images for sham **(A)** and mTBI **(B)**. The intact YFP+ pyramidal neuron sampling distribution (magenta bars) is symmetric with respect to the SCWM. **(C)** Cumulative frequency distribution plot shows a high degree of overlap between sham (gray) and mTBI (gold). **(D)** Quantitative analysis confirmed no sampling difference. Statistics: multilevel analysis with animals as a random variable. Data summarized with means and standard error of the mean (SEM) error bars.

**Figure 6:**
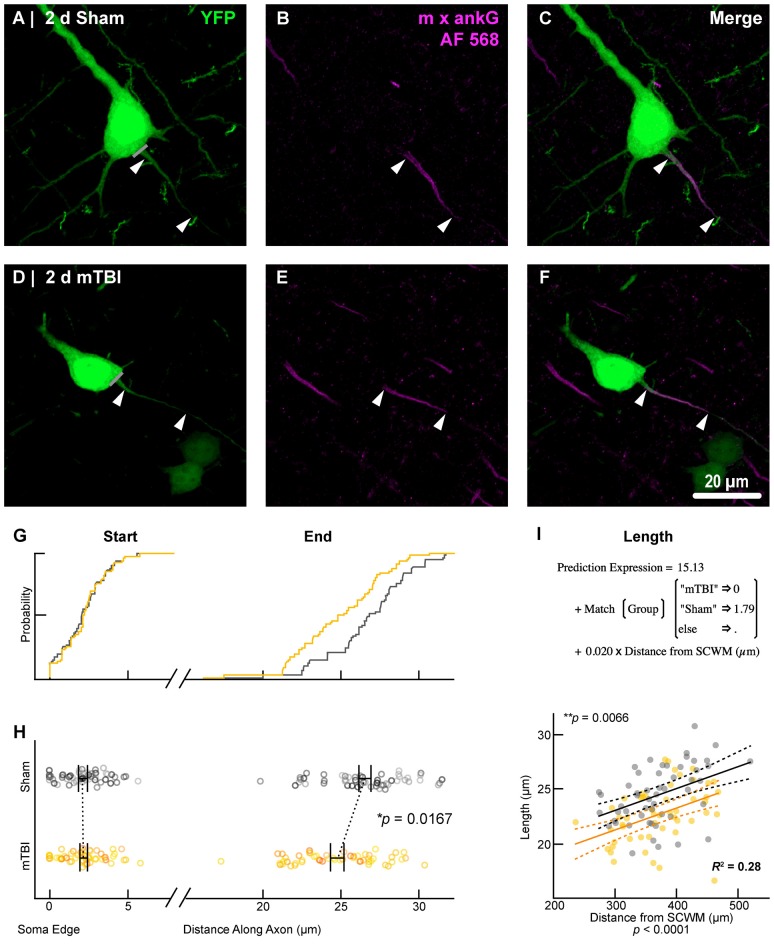
AnkG length decreases from the distal AIS after 2 days post-mTBI. Representative images of sham **(A–C)** and mTBI **(D–F)** of YFP (**A,D**; green) and ankG (**B,E**; magenta). **(C,F)** Composite image showing YFP+ axon colocalization with ankG, demarcating the AIS. YFP traces were taken from the line drawn across the ado-somatic vertex **(A,D)**, through and past the region of the AIS **(C,F)**. **(G–I)** Quantitative analysis of ankG profiles of sham (gray) and mTBI (gold). **(G)** Cumulative frequency distribution plots showing overlap in start position but a proximal shift in mTBI end position. **(H)** Multilevel analysis of start and end positions with animals as a random variable depicted by different shades. Data summarized with means and SEM error bars. Note that there is no change in ankG start position; however, there was a significant decrease in the end position. This resulted in an overall decrease in length from the distal AIS. **(I)** Multilevel analysis of ankG length with animals as a random variable and distance from SCWM as a covariate. Plot of ankG length as a function of distance from SCWM (solid lines) with 95% confidence intervals (CI; dashed lines) for sham and mTBI. Note that distance from SCWM is a significant covariate of ankG length, but there is no interaction with experimental groups indicated seen as a parallel shift in lines.

To estimate AIS position and length (Figure 6), we quantified immunofluorescent profiles of ankG, a key structural protein underpinning AIS function (Grubb and Burrone, 2010). Consistent with previous reports from our laboratory and others (Greer et al., 2013; Gutzmann et al., 2014; Clark et al., 2016), ankG labeling throughout the neocortex was restricted to the AIS and nodes of Ranvier (Figure 3B). Importantly, ankG labeling of intact YFP+ pyramidal neurons clearly delineated the AIS in sections from both sham and mTBI mice (Figures 6B,E). For ankG multilevel modeling, intact YFP+ pyramidal neuron summary of outcome measures (mean ± SEM) and statistics were calculated from nested data sets (sham: *N* = 53 AIS total, 10–11 AIS nested per animal, *n* = 5 mice; mTBI: *N* = 54 AIS total, 5–13 AIS nested per animal, *n* = 6 mice). The sampling distribution of intact YFP+ pyramidal neurons with respect to distance from the SCWM interface (Figure 5) was symmetrical between groups (sham = 372 ± 14 μm, mTBI = 368 ± 14 μm; *t*_8.0_ = 0.18, *p* = 0.8585). Within this same data set (Figure 6), the ankG start position with respect to soma of origin was similar between groups (sham = 2.1 ± 0.16 μm, mTBI = 2.2 ± 0.16 μm; *t*_6.41_ = −0.50, *p* = 0.6342). In contrast, we observed a statistically significant decrease by 6.8% (−1.8 ± 0.59) in ankG end position (sham = 26.6 ± 0.42 μm, mTBI = 24.8 ± 0.42 μm; *t*_7.4_ = −3.08, *p* = 0.0167). Overall, this resulted in ankG length significantly decreasing by 7.8% (−1.9 ± 0.58 μm) from the distal end (sham = 24.4 ± 0.41 μm, mTBI = 22.5 ± 0.41 μm;* t*_7.7_ = −3.30, *p* = 0.0114; *R*^2^ = 0.16). Because layer 5 pyramidal neurons are heterogeneous (Chagnac-Amitai et al., 1990; Hattox and Nelson, 2007; Le Bé et al., 2007), varying structurally and functionally with cortical depth (e.g., layer 5a vs. 5b), we controlled for this potential confound in analyzing plasticity (Jacob et al., 2012) by measuring the AIS distance from SCWM. Multilevel analysis (*N* = 107 AIS total) revealed that distance from SCWM is a significant covariate (*d*_SCWM_) of both ankG end position (*t*_88.0_ = 4.07, *p* < 0.0001) and overall length (*t*_79.0_ = 4.52, *p* < 0.0001). Specifically, ankG end position (*d*_SCWM_ = 0.019 ± 0.005) and length (*d*_SCWM_ = 0.020 ± 0.004) are directly proportional to distance from SCWM. The equal slopes of the best-fit lines for ankG length as a function of distance from SCWM (Figure 6I) indicated that there was no interaction with experimental groups (end position: *t*_86.0_ = −0.82, *p* = 0.3120; length: *t*_80.3_ = 0.69, *p* = 0.5512). Consistent with the symmetrical sample distribution intact YFP+ pyramidal neurons in layer 5 S1BF (Figure 5), the statistical model yielded similar values after leveraging, with respect to distance from SCWM, ankG end position (sham = 26.6 ± 0.36 μm, mTBI = 24.9 ± 0.39 μm) and length (sham = 24.4 ± 0.33 μm, mTBI = 22.6 ± 0.35 μm). Additionally, the leveraged ankG length after mTBI was significantly shorter by 7.4% (−1.8 ± 0.48 μm) compared to shams (*t*_7.4_ = −3.74, *p* = 0.0066; *R*^2^ = 0.28). Notably, accounting for this covariate resulted in 20% decrease in SEM (0.41–0.33) and a 75% increase in the *R*^2^ (0.16–0.28). Hence, while this reduction in ankG length is relatively modest, multilevel modeling revealed that mTBI accounted for almost 30% of the variability between data sets, and the effect size was robust (Cohen’s *d* = 0.76). Further, ankG was shortened from the distal AIS microdomain that contains the “trigger zone” for AP firing, where a highly dense distribution of NaV1.6 sets the threshold for AP initiation (Kole et al., 2008).

**Figure 7:**
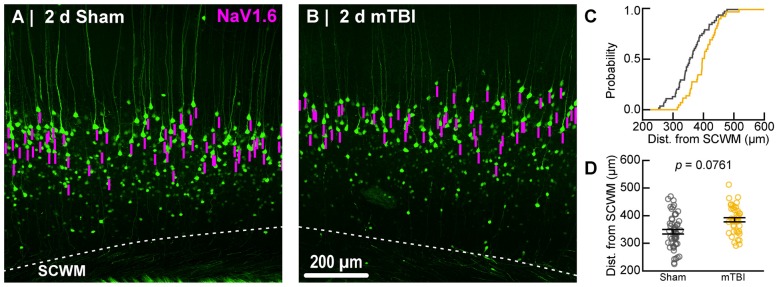
Sampling distribution of intact YFP+ pyramidal neurons for NaV1.6 quantitative analysis. **(A,B)** Superimposed maximum intensity projections of 10× overview images for sham **(A)** and mTBI **(B)**. The intact YFP+ pyramidal neuron sampling distribution (magenta bars) with respect to the SCWM appeared slightly asymmetric, with mTBI having several samples located more superficially (upper right aspect). **(C)** Cumulative frequency distribution plot showing mTBI (gold) samples are shifted toward increased distance from SCWM compared to sham (gray). **(D)** Quantitative analysis confirmed revealed there was no statistically significant difference in sampling. Statistics: multilevel analysis with animals as a random variable. Data summarized with means and SEM error bars.

**Figure 8:**
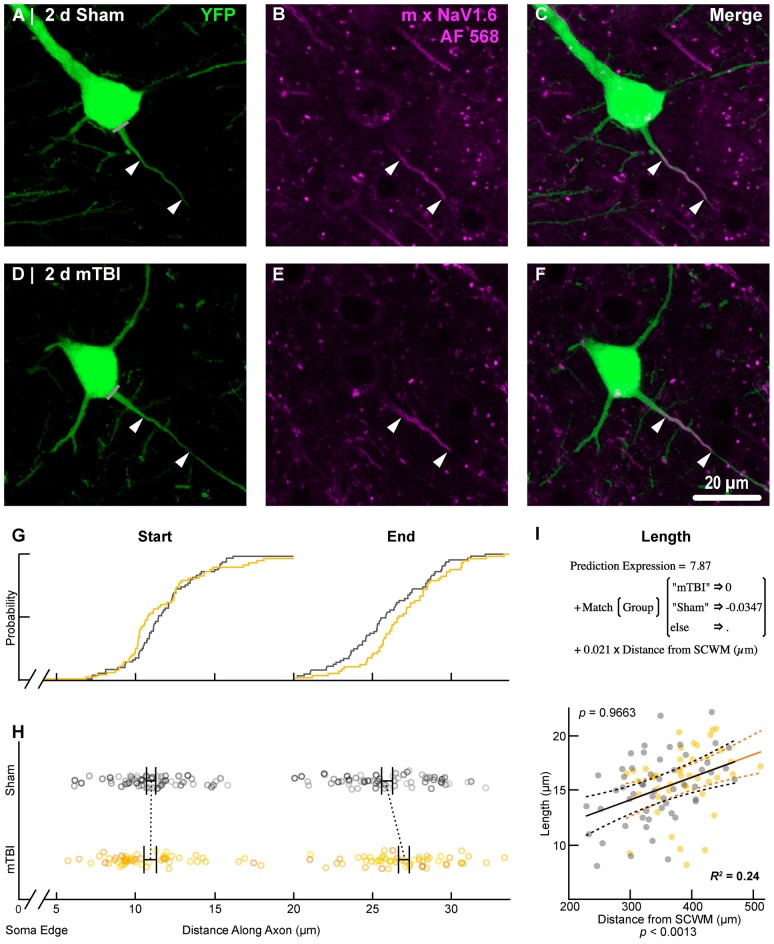
NaV1.6 geometry does not change after mTBI. Representative images of sham **(A–C)** and mTBI **(D–F)** of YFP (**A,D**; green) and NaV1.6 (**B,E**; magenta). **(C,F)** Composite image showing YFP+ axon colocalization with NaV1.6, demarcating the distal AIS. **(G–I)** Quantitative analysis of NaV1.6 profiles of sham (gray) and mTBI (gold). **(G)** Cumulative frequency distribution plots showing overlap in start position but a distal shift in mTBI end position. **(H)** Multilevel analysis of start and end positions with animals as a random variable depicted by different shades. Data summarized with means and SEM error bars. Note that there was no change in NaV1.6 start or end position. **(I)** Multilevel analysis of NaV1.6 length with animals as a random variable and distance from SCWM as a covariate. Plot of NaV1.6 length as a function of distance from SCWM (solid lines) with 95% CI (dashed lines) for sham and mTBI. Distance from SCWM is a significant covariate of NaV1.6 length. This accounted for the overlap in sham and mTBI plots compared to the shift seen in the uncorrected distribution of the end position **(G)**.

To estimate the distal AIS “trigger zone” position and length (Figure 8), we quantified immunofluorescent profiles of NaV1.6. Consistent with previous reports from other labs (Lorincz and Nusser, 2008a; King et al., 2014), NaV1.6 labeling throughout the neocortex was restricted to the distal AIS and nodes of Ranvier (Figure 3C). Importantly, NaV1.6 labeling of intact YFP+ pyramidal neurons clearly delineated the distal AIS in sections from both sham and mTBI mice (Figures 8B,E). For NaV1.6 multilevel modeling, intact YFP+ pyramidal neuron summary of outcome measures (mean ± SEM) and statistics were calculated from nested data sets (sham: *N* = 54 AIS total, 7–14 AIS nested per animal, *n* = 5 mice; mTBI: *N* = 44 AIS total, 5–9 AIS nested per animal, *n* = 6 mice). The sampling distribution of intact YFP+ pyramidal neurons with respect to distance from the SCWM interface (Figure 7) was not statistical different between groups (sham = 339 ± 16 μm, mTBI = 384 ± 15 μm; *t*_9.55_ = 1.99, *p* = 0.0761). Within this same data set (Figure 8), the NaV1.6 start position with respect to soma of origin was similar between groups (sham = 11.0 ± 0.28 μm, mTBI = 11.0 ± 0.32 μm; *t*_5.3_ = 0.12, *p* = 0.8708). Unlike our ankG findings at the distal AIS, we did not observe a statistically significant difference in NaV1.6 end position (sham = 26.0 ± 0.61 μm, mTBI = 27.0 ± 0.59 μm; *t*_9.7_ = 1.20, *p* = 0.2876) or overall length (sham = 15.0 ± 0.60 μm, mTBI = 16.0 ± 0.60 μm; *t*_9.9_ = 1.24, *p* = 0.2448).

In line with our ankG findings (Figure 6I), multilevel analysis also revealed that distance from SCWM is a significant covariate of both NaV1.6 end position (*t*_71.7_ = 3.80, *p* = 0.0003; *d*_SCWM_ = 0.020 ± 0.005) and NaV1.6 length (*t*_59.9_ = 3.38, *p* = 0.0013; *d*_SCWM_ = 0.021 ± 0.006; Figure 8I). Notably, the coefficient of distance from SCWM (*d*_SCWM_) was remarkably similar between ankG and NaV1.6 for both end position and length (average = 0.02, 95% CI: 0.018–0.021; *n* = 4). This observations provided further evidence supporting that AIS length is directly proportional to distance from SCWM, wherein upper vs. lower pyramidal neurons have longer and shorter AIS, respectively. Specifically, the statistical model predicts AIS length from distal end increases ~2 μm for every 100 μm from SCWM (Figures 6I, 8I). Considering layer 5 S1BF is ~300 μm thick (Figures 5, 7), this corresponds to ~6 μm difference in AIS length from the distal end between upper and lower pyramidal neuron populations. While the sampling distributions with respect to the distance from SCWM interface between sham and mTBI were not significantly different (as described above), in light of this covariate’s magnitude, we compared the means of non-leveraged vs. leveraged with respect to distance from SCWM of NaV1.6 end position and length using the Wilcoxon signed-rank test. For sham data (*n* = 5 mice), including the distance from SCWM covariate in our multilevel model did result in a statistically significant difference between the means NaV1.6 end position (leveraged = 26.1 ± 0.52 μm;* X*^2^ = −2.5, *p* = 0.6250) or length (leveraged = 15.4 ± 0.55 μm;* X*^2^ = −6.5, *p* = 0.1250). In contrast, for mTBI data (*n* = 6 mice) the distance from SCWM covariate in the multilevel model yielded significantly decreased mean values for both NaV1.6 end position (leveraged = 26.2 ± 0.52 μm; *X*^2^ = 10.5, *p* = 0.0313) and length (leveraged = 15.4 ± 0.56 μm; *X*^2^ = 10.5, *p* = 0.0313). This difference in NaV1.6 end position (−0.70 ± 0.38) and length (−0.58 ± 0.42) from the distal AIS is consistent with our model’s predicted effect of a −45 μm difference between sham (339 μm) and mTBI (384 μm) mean distance from SCWM (e.g., 0.02 × −45 μm = −0.9 μm; Figures 7C,D).

Collectively, these data suggest disparate responses between ankG and NaV1.6 at the distal AIS 2 days after mTBI. Restructuring our multilevel model by changing the experimental condition to AIS component allowed us to directly assess for differences between ankG and NaV1.6 end position. As described above, we accounted for nested measurements per animal. Additionally, in this multilevel model we also accounted for the dependency resulting from sampling two sections per animal for single labeling of ankG and NaV1.6. To determine if there was a differential response at the distal AIS, we compared the leveraged means of ankG and NaV1.6 end positions in both sham and mTBI data sets. In shams (*n* = 5 mice), the leveraged end position for ankG (26.3 ± 0.55 μm; *N* = 53 AIS, 10–11 AIS nested per animal) and NaV1.6 (26.0 ± 0.55 μm; *N* = 54 AIS, 7–14 AIS nested per animal) were similar (*t*_8.2_ = 0.47, *p* = 0.6522; *N* = 10 sections, 2 per animal). Conversely, after mTBI (*n* = 6 mice) the leveraged end position for ankG (25.0 ± 0.34 μm; *N* = 54 AIS, 5–13 AIS nested per animal, 54 AIS total) was less distal than NaV1.6 (26.5 ± 0.37 μm; *N* = 44 AIS, 5–9 AIS nested per animal). This difference of 5.6% (–1.48 ± 0.50) between ankG and NaV1.6 end positions was statistically significant (*t*_8.6_ = −2.94, *p* = 0.0172; *N* = 12 sections, 2 per animal). Overall, the multilevel model accounted for a large proportion of the variability between data sets (*R*^2^ = 0.23) and the effect size was substantial (Cohen’s *d* = 0.61). Taken together, these data show that ankG structural plasticity is uncoupled from NaV1.6 at 2 days post-mTBI. Further, these data provide compelling evidence for S1BF layer 5 pyramidal neuron AIS length as a function of distance from the SCWM. Lastly, these findings reveal that AIS length both after mTBI and as a function of distance from the SCWM is modulated at the site of AP initiation that is regulated by NaV1.6.

#### Perisomatic GABAergic Bouton Density

**Figure 9:**
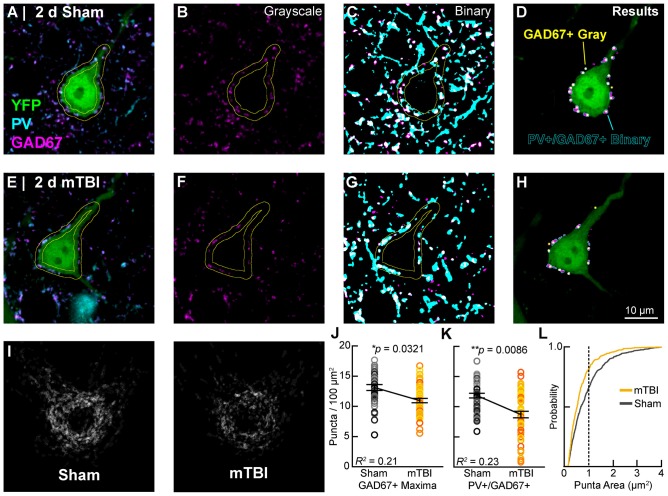
Intact YFP+ pyramidal neuron perisomatic GABAergic bouton density decreases 2 days post-mTBI. Representative images of sham **(A–D)** and mTBI **(E–H)** glutamate decarboxylase-67 (GAD67+; **B,F**; magenta) and parvalbumin (PV+; **A,C,E,G**; cyan) punch along the perimeter of YFP+ pyramidal neurons (**A,E,D,H**; green). Perisomatic bouton density was quantified in 8-bit gray scale images of GAD67 expression **(B,F,J)** and also binary images of colocalized PV+/GAD67+ puncta **(C,G,I,K,L)**. **(D,H)** Outcome of automated quantification using gray scale and binary images was comparable. Perisomatic GAD67 expression (magenta) and result of gray scale analysis (yellow dots) largely overlapped with results of PV+/GAD67+ binary image analysis (cyan outlines). **(I)** A qualitative decrease in perisomatic GABAergic bouton density is seen in superimposed images of binary PV+/GAD67+ profiles. **(J,K)** Multilevel analysis with animals as a random variable depicted by different shades for sham (gray) and mTBI (gold/orange). Data summarized with means and SEM error bars. GAD67+ maxima in gray-scale images and PV+/GAD67 puncta in binary images were both significantly reduced in mTBI vs. sham. The absolute values as well as relative decrease were similar between gray-scale and binary image analyses. **(L)** Cumulative frequency distribution of PV+/GAD67+ puncta area. Vertical line at 1 μm^2^ corresponds to average size of a GABAergic presynaptic bouton. This shift in the mTBI plot shows that PV+/GAD67+ puncta approximating the average area of a GABAergic bouton accounting for the majority of the loss.

In concert with intrinsic/postsynaptic AIS structural plasticity, we observed a loss of GABAergic inputs along the perisomatic domain of intact YFP+ pyramidal neurons in layer 5 S1BF, revealing evidence for extrinsic/presynaptic change 2 days after mTBI (Figure 9). Immunolabeling GAD67, the GABA-synthesizing enzyme enriched in PV+ presynaptic boutons (Fish et al., 2011) allowed us to conduct a high precision quantitative analysis of the major source of inhibitory input at the perisomatic domain, which strongly regulates AIS activity. We quantified perisomatic GABAergic bouton density of only intact YFP+ pyramidal neurons in S1BF using the same sampling method for AIS studies (Figures 5, 7). For perisomatic GABAergic bouton density (puncta/100 μm^2^) multilevel modeling, intact YFP+ pyramidal neuron summary of outcome measures (mean ± SEM) and statistics were calculated from nested data sets (sham: *N* = 48 somas total, 5–13 somas nested per animal, *n* = 5 mice; mTBI: *N* = 50 somas total, 5–14 somas nested per animal, *n* = 5 mice). Using two different quantitative methods, we observed a decrease in perisomatic GABAergic bouton density in both GAD67+ (Figures 9B,F,J) and GAD67+/PV+ (Figures 9C,G,K,L) immunoreactive profiles 2 days after mTBI. Specifically, the density of GAD67+ profiles in gray-scale images between sham (13.0 ± 0.57 puncta/100 μm^2^) and mTBI (10.9 ± 0.57 puncta/100 μm^2^) were significantly different; *t*_7.8_ = −2.61, *p* = 0.0321). Similarly, the density of GAD67+/PV+ profiles in binary images between sham (12.0 ± 0.55 puncta/100 μm^2^) and mTBI (8.7 ± 0.56 puncta/100 μm^2^) were also significantly different; *t*_5.4_ = −4.04, *p* = 0.0086). The magnitudes of change were comparable between gray and binary quantitative methods, with intact YFP+ pyramidal neuron perisomatic GABAergic bouton density decreasing by 16% and 24%, respectively. Additionally, the effect sizes were robust for both methods (Cohen’s *d*: gray-scale = 0.71; binary = 0.99). Lastly, analysis of the cumulative distribution plot of the size of PV+/GAD67+ puncta (segmented particles in binary images) revealed that the greatest decrease occurred in particles that were the average size (~1 μm^2^) of GABAergic puncta (Figure 9L). Taken together, these data provide substantial evidence for diffuse presynaptic GABAergic terminal loss 2 days after mTBI.

#### AIS Functional Plasticity

**Figure 10:**
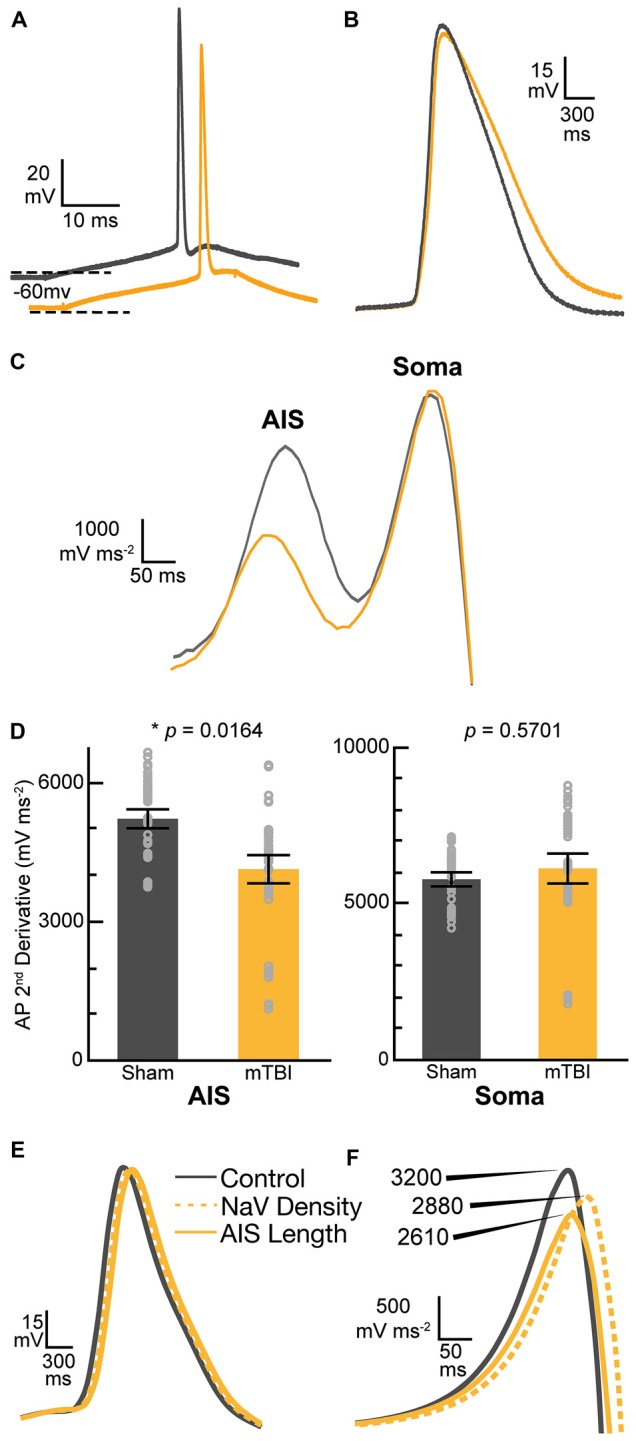
Action potential (AP) acceleration at the AIS is reduced 2 days post-mTBI. **(A–D)** Whole-cell patch-clamp analysis of intact YFP+ pyramidal neurons of layer 5 primary somatosensory barrel field (S1BF) in sham (gray) and mTBI (gold) *ex vivo* live slices. **(A)** Representative traces of AP recorded at somas. The mTBI trace is offset for better comparison. Dashed lines indicate holding potential. **(B)** Enlarged traces from **(A)** showing AP waveform. Note slightly longer AP decay after mTBI. **(C)** Plot of second derivative of the membrane voltage. The first and second peak amplitudes were used to compare AP acceleration at the AIS and soma, respectively. **(D)** Multilevel analysis of the second derivative of membrane voltage (AP acceleration) with animals as a random variable and accounting for the five recorded AP nested within each cell. Data summarized with means and 95% CI error bars. The peak amplitude at the AIS was significantly reduced, reflecting attenuated AP acceleration. In contrast, there was no change in the AP acceleration at the soma. **(E,F)** Computational modeling of a layer 5 pyramidal neuron. Evoked AP waveforms in simulations **(E)** using experimentally observed AIS lengths were similar to AP recorded from intact YFP+ pyramidal neurons **(A)**. Overall, changing AIS length or NaV density at the distal AIS by 7% did not have an overt effect on AP waveform. **(F)** The plots of computationally derived second derivatives of membrane voltage were also comparable to experimental observations **(B)**. Modeling data showed that a 7% decrease in AIS length or NaV1.6 density at the distal AIS both decrease peak amplitude of the second derivative plot, consistent with attenuated AP acceleration.

Our findings of presynaptic input loss in concert with postsynaptic AIS remodeling have been associated with alterations of neuronal excitability in sensory circuits reported by others (Kuba et al., 2010, 2014). Appreciating the known intrinsic properties of the AIS and the mechanisms that modulate its structure and function (Bender and Trussell, 2012), we followed our morphological studies with electrophysiological assessments at 2 days post-mTBI. Specifically, we employed whole-cell patch-clamp analysis of intact YFP+ pyramidal neurons in layer 5 S1BF to compare intrinsic AIS functional properties between sham (*n* = 13 cells from 3 mice; 4–5 AP nested per cell, *N* = 63 AP total) and mTBI (*n* = 14 cells from 4 mice; 5 AP nested per cell, *N* = 70 AP total) groups using multilevel modeling. Similar to shams, current injection evoked an AP in all patched intact YFP+ pyramidal neurons after mTBI, consistent with the diffuse and subtle nature of mTBI pathophysiology. While AP waveforms were comparable between sham and mTBI (Figures 10A,B), AP decay tau significantly decreased by 79% after mTBI (*p* = 0.0014; Table 1). Additionally, we found that AP threshold was ~3 mV more depolarized after mTBI (−46.2 ± 1.0 mV) compared to shams (−49.6 ± 1.0 mV). This difference in AP threshold was statistical significant (*t*_25_ = 2.41, *p* = 0.0236; Cohen’s *d* = 0.95), and consistent with altered AIS intrinsic function. While AP initiation occurs at the AIS, after spiking the signal travels both anterograde down the axon, and retrograde toward the soma. Therefore, AP recorded by patching the cell body reflects a combination of AP generation at the AIS and back-propagation in the soma (Hu et al., 2009). To further dissect intrinsic AIS function from patch-recordings at the cell body, we calculated the second derivative of membrane voltage (i.e., AP acceleration), which showed two peaks (Figure 10C). In the plot of the second derivative, the amplitude of the first and second peaks corresponded to AP acceleration at the AIS and soma, respectively (Khaliq and Raman, 2006; Meeks and Mennerick, 2007). Interestingly, multilevel modeling revealed AP acceleration is significantly reduced at the AIS (*t*_25_ = −2.57, *p* = 0.0164), but not the soma (*t*_25_ = 0.58, *p* = 0.5701; Figures 10C,D). Specifically, AP acceleration at the AIS decreased by 21% after mTBI (4104 ± 296 mV/μs^2^) compared to shams (5203 ± 308 mV/μs^2^), and the effect size was large (Cohen’s *d* = 1.01). AP threshold in pyramidal neurons is set by NaV1.6 channels localized at the distal AIS (Kole et al., 2008). These data showing AP threshold depolarization and attenuation of AP acceleration specifically at the AIS are consistent with alteration of NaV1.6 membrane distribution after mTBI. Together, our morphological and electrophysiological studies provide significant evidence of intact YFP+ pyramidal neuron AIS structural and functional plasticity after mTBI.

**Table 1:** Intrinsic action potential (AP) properties of intact yellow fluorescent protein (YFP+) pyramidal neurons 2 days postinjury.

AP Property	Sham	mTBI	*t*_25_ ratio	*p*-value
Amplitude (mV)	108 ± 4.5	106 ± 4.4	–0.25	0.8039
Half-width (ms)	0.74 ± 0.040	0.81 ± 0.038	1.30	0.2048
Rise Tau (ms)	0.23 ± 0.115	0.44 ± 0.111	1.34	0.1933
Decay Tau (ms)	6.3 ± 1.03	1.2 ± 0.99	–3.6	0.0014

*Data summarized as mean ± SEM. Statistics: multilevel analysis of group and animals with five AP nested in each cell. Sham n = 13 cells from three mice (63 AP, 2 missing) and mTBI n = 14 cells from four mice (70 AP total)*.

#### Layer 5 Pyramidal Neuron Modeling

While AIS excitability varies with axial geometry such as length (Kuba et al., 2010), voltage-gated ion channel density is also a critical determinant of intrinsic AP properties (Kole et al., 2008). To investigate the functional consequences of AIS structural plasticity and explore the effect varying NaV1.6 density we used an anatomically and physiologically accurate computational model of a layer 5 pyramidal neuron (Hallermann et al., 2012). This computational model allowed us to probe AP dynamics specifically at the AIS. The AP waveforms in the model (Figure 10E) were qualitatively similar to those observed experimentally (Figure 10B). Comparing the effect of AIS length differences based on our experimental observations of ankG immunofluorescent profiles, we found that shortening the length by 7% resulted in a 19% decrease in the peak of the second derivative of membrane voltage, reflecting attenuated AP acceleration (Figure 10F). To help elucidate potential functional consequences of ankG and NaV1.6 uncoupling we evaluated the effect of a proportionate decrease in NaV density at the distal AIS. Without changing AIS length, a 7% decrease in NaV density at the distal AIS also attenuated AP acceleration by 10%. Thus, AP kinetics at the AIS depends on both geometry and density of functional components. Collectively, these morphological and computational findings suggest that even subtle AIS changes may alter AP spike dynamics among intact pyramidal neurons after mTBI.

## Discussion

This study provides compelling evidence of intact pyramidal neuron AIS structural and functional plasticity after clinically relevant mTBI in mice. Consistent with human mTBI, midline cFPI reproducibly evoked DAI without mass lesions, neocortical contusion, and/or overt cell death (Mittl et al., 1994; Saatman et al., 2008; Andriessen et al., 2010; Bigler and Maxwell, 2012; Yuh et al., 2013; Shultz et al., 2016). Exploiting restricted YFP expression in a discrete subset of layer 5 pyramidal neurons and an established AIS marker (ankG), we confirmed subcellular structural plasticity within the non-DAI/intact population. In this mTBI-mediated response, the loss of presynaptic GABAergic boutons surrounding the perisomatic domain of intact pyramidal neurons suggested compromised AIS inhibition mediated by PV+ interneuron input. Consistent with AIS structural change, patch-clamp recordings of intact YFP+ layer 5 pyramidal neurons revealed attenuated AP acceleration after mTBI. This finding was further supported by simulations using a realistic layer 5 pyramidal neuron model that showed even a subtle decrease in AIS length or NaV1.6 density attenuates AP acceleration. Taken together, these findings demonstrate significant structural and functional changes of both intrinsic and extrinsic/synaptic components that are major regulators of AIS activity. The conclusions of this study depart from current thought on mTBI, which has been based primarily on the role of structural disconnection of white matter tracts (Sharp and Ham, 2011; Sharp et al., 2014). Collectively, the novel subcellular and neurophysiologic substrates identified in this study significantly extend our knowledge of neocortical circuit dysfunction following mTBI.

### cFPI: Clinical Relevance and Utility for Evaluating Neocortical Circuit Dysfunction

Several features of our cFPI model underscore its clinical relevance and utility for evaluating both DAI and intact neuronal populations in neocortical circuits after mTBI. We used loss of righting reflex in mice as a surrogate for loss of consciousness in humans to assess injury severity, which had a similar duration ranging 3–5 min (Shaw, 2002; Malec et al., 2007; Schrader et al., 2009). While this was our only behavioral assessment, we monitored arterial oxygen saturation, heart rate, RPM and core body temperature in all mice used for structural and functional studies. Specifically, all mice were in physiological homeostasis. Importantly, evaluating these parameters help distinguish our work from most other investigators who do not perform routine physiologic monitoring to exclude secondary insults, which could severely confound the assessment of network disruption via diffuse structural and functional changes.

Capitalizing on restricted neuronal labeling with YFP including the total axonal length, readily traceable within the neocortical gray, allowed the unequivocal identification of mTBI-induced DAI vs. non-DAI/intact populations in both fixed tissue sections and live slices. Specifically, we readily identified intact pyramidal neurons by following their YFP+ axons from the soma of origin to the SCWM interface. Previously, we reported that mTBI induces DAI primarily within the AIS of the YFP+ layer 5 pyramidal neurons (Greer et al., 2013). Additionally, in a former publication we showed that the most commonly used DAI marker (β-amyloid precursor protein) did not colocalize with YFP axons in either the SCWM or corpus callosum (Hånell et al., 2015b). Taken together, these findings strongly support the premise that virtually all YFP+ pyramidal neuron DAI occurs within the neocortical gray. Based on this premise, intact YFP+ pyramidal neurons vs. those undergoing DAI can be reliably identified by examining their axonal segments spanning neocortical layers 5/6 to the SCWM interface.

Pyramidal neurons are the major excitatory component in cortical networks (Fairen et al., 1984; DeFelipe and Fariñas, 1992; Somogyi et al., 1998). Previously we showed that intact YFP+ pyramidal neurons intrinsic and extrinsic/synaptic properties were altered post-mTBI and developed over time (Greer et al., 2012; Hånell et al., 2015a; Sun and Jacobs, 2016). Initially after mTBI, the intrinsic properties of intact pyramidal neurons paralleled that found in the DAI population, suggesting that ion and/or neurotransmitter imbalance had an indiscriminant overriding effect on currents (Greer et al., 2012). However, by 2 days post-mTBI a functional divergence was identified wherein the intact YFP+ pyramidal neuron population showed distinct electrophysiological changes implicating AIS structural-functional alteration. These changes occurred in parallel with overall increased network excitability (Hånell et al., 2015a), implicating loss of inhibition (Isaacson and Scanziani, 2011; Yizhar et al., 2011; Lazarus et al., 2015). Collectively, these previous findings by our laboratory formed the basis of the current study focusing on the effect of mTBI on intrinsic structure and function of the AIS as well as the extrinsic/synaptic GABAergic inputs that control its output (Cobb et al., 1995; Pouille and Scanziani, 2001; Freund, 2003; Klausberger et al., 2003; Klausberger and Somogyi, 2008; Cardin et al., 2009; Atallah et al., 2012; Pouille et al., 2013; Lazarus et al., 2015; Wefelmeyer et al., 2015).

Building upon our previous studies in YFP-H mice, we succeeded in conducting a highly detailed analysis within this specific subset of neocortical layer 5 intact pyramidal neurons showing no evidence of DAI. This was a crucial component of our study since the AIS ion channel distribution differs across neuronal subtypes (Lorincz and Nusser, 2008a), and even within a neuronal subtype (Hu et al., 2009). Additionally, as seen shown in the current study, there is substantial variability in position and length. Further, because DAI affects only a small fraction of neurons within a pyramidal neuron population (Greer et al., 2011) that represent 80% of all neocortical neurons (DeFelipe and Fariñas, 1992), quantification of such heterogeneous population (Molnár and Cheung, 2006) remains an important confounder. Our previous reports underscore this issue, showing that the neocortical pyramidal neuron AIS has a predilection for DAI (Greer et al., 2013). In the current study we show DAI occurring within the AIS severely disrupts ankG and NaV1.6 expression (Figure 4). Hence, even a 5% burden of DAI (Greer et al., 2011) causing AIS disruption would substantially contribute to data variability.

### Layer 5 Pyramidal Neuron AIS “Tuning”

To the best of our knowledge, this is the first study to account for these issues by examining only intact, Thy1-expressing, layer 5 pyramidal neurons. The important distinction in the current report is that we detected a decrease in AIS length within 2 days after cFPI-induced mTBI specifically within the layer 5 pyramidal neuron populations with no overt structural axonal abnormalities and/or evidence of DAI. For structural studies, we relied on immunofluorescent labeling of ankG and NaV1.6 as surrogate markers of the AIS and associated “trigger zone” at the distal end, respectively (Grubb et al., 2011; Kole and Stuart, 2012). An unexpected finding in the current study that followed from our *a priori* control parameters was that distance from SCWM was a significant covariate of AIS length (Figure 6I). Specifically, intact YFP+ pyramidal neurons AIS length from the distal end varied within the ~300 μm span of layer 5 neocortex. Importantly, this pattern was observed in both ankG and NaV1.6 assessments, which yielded virtually the same coefficient of 0.02 in each of the statistical models. This observed variance in AIS structure is consistent with known properties of layer 5 pyramidal neurons which are subdivided into layer 5a and 5b and populated by multiple subtypes characterized by different morphological and electrophysiological features (Chagnac-Amitai et al., 1990; Schubert et al., 2001; Hattox and Nelson, 2007). Additionally, the novel findings in this study are consistent with the “fine-tuning” of the AIS previously described in visual (Gutzmann et al., 2014), as well as auditory circuits (Kuba et al., 2010; Kuba, 2012). Specifically, Gutzmann et al. (2014) showed AIS length from the distal end varies between layer 2/3 and layer 5, representing functionally distinct regions within a neocortical column (Mountcastle, 1979; Somogyi et al., 1998; DeFelipe et al., 2003). Building on this previous work showing inter-laminar differences, in the current report we demonstrate intra-laminar AIS variability, consistent with the presence of layer 5 pyramidal neuron subpopulations (Molnár and Cheung, 2006). While AIS varying as a function of neocortical depth was observed in both 2 days sham-injury and mTBI mice, the lack of interaction between distance from SCWM and experimental groups supports that this finding reflects a novel AIS property with important basic science implications beyond the scope of our injury paradigm.

### Uncoupling of ankG and NaV1.6 after mTBI

The current report highlights the importance of measuring both AIS proximal and distal positions with respect to soma of origin. Since structural remodeling can occur at the start and/or end of the AIS (Grubb and Burrone, 2010; Kuba et al., 2010; Evans et al., 2013, 2015), knowledge of AIS position with respect to soma of origin is an important metric. This issue becomes even more critical when factoring proximal and distal AIS subdomains are populated by different ion channel subtypes (Lorincz and Nusser, 2008a; Hu et al., 2009). A key morphological finding in this current study is that while there was no change in ankG position with respect to the soma of origin, the length significantly decreased from the distal end (Figure 6H). This finding adds to the repertoire of studies demonstrating AIS length modulation with altered neuronal activity (Evans et al., 2015), sensory circuit development (Kuba et al., 2010; Gutzmann et al., 2014), models of injury (Baalman et al., 2013; Hinman et al., 2013), and also congenital or acquired diseases (Kaphzan et al., 2011; Hamada and Kole, 2015; Clark et al., 2016).

Surprisingly, we did not observe parallel changes in NaV1.6 length at the distal AIS, the site of AP initiation (Stuart et al., 1997; Palmer and Stuart, 2006; Kole et al., 2008; Popovic et al., 2011). To our knowledge, this is the first report that demonstrates ankG remodeling uncoupled from changes in NaV1.6 geometric distribution. Hence, this observation raises the question whether other AIS components may stabilize NaV1.6 in the absence of ankG. For example, the cell adhesion molecule neurofascin-186, which is known to stabilize the AIS (Ratcliffe et al., 2001; Hedstrom et al., 2007; Zonta et al., 2011; Kriebel et al., 2012). Alternatively, a decrease in ankG length could elicit a subtle decrease NaV1.6 density (Hedstrom et al., 2008; Gasser et al., 2012) in the absence of length change. In such a case, a decrease in ankG length from the distal end resulting in NaV1.6 redistribution may be a potential mechanism underlying AIS functional changes discussed below. On the other hand, NaV1.6 activity and/or association with ankG may be modulated biochemically (Garrido et al., 2003; Bréchet et al., 2008; Hund et al., 2010; Evans et al., 2015). Notably, is the potential involvement of calcineurin, a phosphatase that has been implicated in both AIS plasticity (Evans et al., 2013, 2015) and mTBI axonal pathophysiology (Singleton et al., 2001; Marmarou and Povlishock, 2006; Reeves et al., 2007; Campbell et al., 2012). Ultimately, in addition to this studies limitation of a single time point, our morphological analyses were confined to ankG and NaV1.6. Thus, future studies are needed with more time points that target other AIS structural proteins as well as different voltage-gate ion channels modulating AP dynamics.

### Evidence for AIS Functional Plasticity

Following our structural studies with electrophysiological assessments revealed further evidence for intact YFP+ pyramidal neuron AIS plasticity after mTBI. While AP waveform was not overtly different after mTBI, we did find a significant change in the decay tau. Interestingly, this suggested potential involvement of potassium channels within the AIS, which have been shown to redistribute after sensory deprivation (Kuba et al., 2015) and modulate AP waveform (Kole et al., 2007). Additionally, we observed that the AP threshold was more depolarized after mTBI. Since NaV1.6 channels set AP threshold (Kole et al., 2008), this provided indirect evidence for intrinsic AIS functional plasticity localized at the distal AIS. Notwithstanding the maintenance of NaV1.6 geometry after mTBI, patch-clamp recordings show AP acceleration is attenuated at the AIS but not the soma. Together, these observations lend further support to the supposition that subtler AIS ion channel redistribution and/or post-translation modification may be operant in the absence of morphological changes. Computer simulations using a realistic model of a layer 5 pyramidal neuron supported this premise. Specifically, decreasing either AIS length by experimentally observed values or NaV1.6 density by the same proportion also decreased AP acceleration, consistent with the data reported by Hallermann et al. (2012) in their characterization of this layer 5 pyramidal neuron model. All together, these data reflect slower NaV1.6 activation specifically at the AIS. Based on these collective findings, we now posit that at 2 days post-mTBI the AIS is in a state of flux, wherein the initial ankG structural remodeling precedes the NaV1.6 geometric redistribution that leads to length reduction.

### Diffuse Loss of Perisomatic GABAergic Terminals

Neocortical balance of excitation-inhibition is regulated by PV+ interneurons that are highly interconnected with local pyramidal neurons (Gulyás et al., 1999; Pouille and Scanziani, 2001; Isaacson and Scanziani, 2011; Packer and Yuste, 2011; Hu et al., 2014; Scholl et al., 2015). The proximity of PV+ terminals to the AIS allows for strong inhibitory control over AIS output (Cobb et al., 1995; Klausberger et al., 2003; Freund and Katona, 2007; Atallah et al., 2012; Pouille et al., 2013; Lazarus et al., 2015; Wefelmeyer et al., 2015). Previously, it has been demonstrated that fulminant deafferentation of sensory input can induce AIS structural plasticity associated with altered neuronal excitability (Kuba et al., 2010). In light of the known features of AIS structure-function and that mTBI-induced abnormal network activity is associated with imbalanced excitation-inhibition, we also evaluated extrinsic/synaptic structural changes in concert with our AIS investigations. In the current study, quantitative analysis of layer 5 intact YFP+ pyramidal neuron perisomatic GAD67+ and PV+/GAD67+ puncta revealed a significant decrease in density. While determining the actual functional consequences of this structural finding is beyond the scope of the current study, this parallel observation to our AIS findings provides additional evidence that local neocortical disruption is a consistent feature of mTBI. In future studies, it would be important to determine if such loss of presynaptic GABAergic input elicits postsynaptic AIS shortening to decrease pyramidal neuron excitability to restore network homeostasis after mTBI.

While the mechanism underlying PV+ interneuron terminal loss was not critically addressed in the current study, recently we showed for the first time that locally projecting neocortical GABAergic interneurons undergo DAI (Vascak et al., 2017). Specifically, we showed that the majority of GABAergic DAI is represented by the PV+ interneuron subclass. Importantly, PV+ interneuron DAI manifested shortly after mTBI and occurred primarily in the perisomatic/AIS domain, prior to the initial branch point of their expansive axonal arbor. Since PV+ interneurons target hundreds of postsynaptic pyramidal neurons (Woodruff et al., 2010; Packer and Yuste, 2011; Taniguchi et al., 2013; Scholl et al., 2015; Takács et al., 2015), disconnection prior to the initial branch point results in widespread, diffuse inhibitory terminal loss. In our recent study, widespread PV+ axonal debris fields within 1 day post-mTBI indicated diffuse terminal degeneration. Consistent with PV+ interneuron DAI and anterograde deafferentation of postsynaptic target sites, in an earlier report from our laboratory we showed ultrastructural evidence of diffuse terminal degeneration along the perisomatic domain of healthy pyramidal neurons within layer 5 neocortex within 2 days post-mTBI (Singleton et al., 2002). Together, these findings suggest that the observed loss of PV+ terminals along the perisomatic domain of intact YFP+ pyramidal neurons is likely due to DAI-induced deafferentation of target postsynaptic sites.

## Conclusion

The AIS is a highly complex and dynamic domain (Hallermann et al., 2012; King et al., 2014; Evans et al., 2015). Intrinsically linked structure-function relationships between scaffolding proteins and ion channels are a crucial for AIS activity (Zhou et al., 1998; Jenkins and Bennett, 2001; Yang et al., 2007). In this way, the neocortical pyramidal neuron AIS is a major determinant of network function and homeostatic plasticity (Grubb et al., 2011; Bender and Trussell, 2012; Kole and Stuart, 2012). This premise has been evaluated in several studies showing AIS disruption associated with multiple disease states involving an imbalance of network function (Kaphzan et al., 2011; Hinman et al., 2013; Hamada and Kole, 2015; Clark et al., 2016). The findings of this study broaden the cerebral landscape affected by mTBI, emphasizing the role of postsynaptic functional disruption of intact neurons. Based on the current data, we posit that AIS plasticity may constitute a compensatory response to mTBI. However, future studies are needed to determine whether AIS structural-functional plasticity is adaptive or maladaptive, which has important implications for restoring excitatory-inhibitory balance in neocortical circuits disrupted by mTBI.

## Author Contributions

MV, KMJ and JTP contributed to the design of the experiments. MV performed all surgeries and confocal imaging. JS collected all electrophysiological data. Both MV and MB performed statistical data analyses. MV, JS, KMJ and JTP contributed to writing of the manuscript.

## Conflict of Interest Statement

The authors declare that the research was conducted in the absence of any commercial or financial relationships that could be construed as a potential conflict of interest.

## Footnotes

^1^www.random.org

## Abbreviations

aCSF: artificial cerebral spinal fluid
AIS: axon initial segment
ankG: ankyrin-G
AP: action potential(s)
BPM: beats per minute
cFPI: central fluid percussion injury
CI: confidence interval
DAI: diffuse axonal injury
*d*_SCWM_: coefficient of distance from subcortical white mater
FOV: field-of-view
GAD67: glutamate decarboxylase (67 kDa)
Kv1: voltage-gated potassium channel
mTBI: mild traumatic brain injury
NaV: voltage-gated sodium channel
PBS: phosphate buffered saline
PV: parvalbumin
ROI: region-of-interest
RPM: respirations per minute
S1BF: primary somatosensory barrel field
SCWM: subcortical white matter
SD: standard deviation
SEM: standard error of the mean
TBI: traumatic brain injury
YFP: yellow fluorescent protein.
